# Decoding Silent Signals: A Systematic Review of ECG and the Quest for an Early Diagnosis of Cirrhotic Cardiomyopathy

**DOI:** 10.7759/cureus.89871

**Published:** 2025-08-12

**Authors:** Sanathanan Neelakantan Ramaswamy, Samyuktha Harikrishnan, Nehal Bhatt, Yashasvi Agarwal, Shalvin Chand, Manvitha Bendagiri Matam, Lubna Mohammed

**Affiliations:** 1 Medicine, Government Erode Medical College and Hospital, Perundurai, IND; 2 Medicine, Gulf Medical University, Ajman, ARE; 3 Medicine, Pramukhswami Medical College, Karamsad, IND; 4 Internal Medicine, Sir H. N. Reliance Foundation Hospital and Research Centre, Mumbai, IND; 5 Medicine, Jawaharlal Nehru Medical College, Belagavi, IND; 6 Medicine, University of Fiji, Lautoka, FJI; 7 Internal Medicine, Gandhi Medical College, Secunderabad, IND; 8 Internal Medicine, Dr VRK Women’s Medical College &amp; Research Centre, Aziznagar, IND

**Keywords:** cardiac markers, cardiomyopathy, cirrhosis, decompensated liver disease, ecg

## Abstract

Cirrhotic Cardiomyopathy (CCM) is a well-known cardiac complication with a high incidence of underdiagnosis by healthcare systems in patients with advanced liver disease. While most of the manifestations of cirrhosis stay within the liver function affected, evidence suggests systemic pathology with myocardial dysfunction. Noninvasive ECG markers of potential CCM include QT interval prolongation, QRS hyper-voltage, and T-wave abnormalities. However, the relationship between ECG abnormalities, structural and functional changes in the heart has not been fully described. This systematic review follows Preferred Reporting Items for Systematic Reviews and Meta-Analyses (PRISMA) 2020 reporting guidelines, providing a literature search of ECG changes and evaluation in patients with cirrhosis to establish their diagnostic and predictive value. A structured search of major literature databases showed eight high-quality studies that met the criteria for inclusion in the review.

QT prolongation has been associated with the severity of the liver disease, as represented by the clinical scoring system. Changes to the myocardial structure lead to the modification of QRS voltage levels and T-wave configuration, as well as increased arrhythmia risk. Variable sensitivity and specificity were found in diagnosing ventricular function with different echocardiographic parameters, leaving the need for multiple types of diagnostics to provide an accurate evaluation of ventricular function. Identification of ECG abnormalities in different stages of CCM is needed for an early detection system that will result in a higher quality of patient care. Evaluating ECG markers, combined with echocardiography data, N-terminal pro-B-type natriuretic peptide (NT-proBNP), and cardiac troponin biomarkers, leads to improved CCM diagnosis and risk stratification for liver transplant patients. Longitudinal research combined with AI-assisted ECG should be the primary focus in future research for standardization of CCM diagnosis and clinical care.

## Introduction and background

Cirrhotic Cardiomyopathy (CCM) represents a heart condition that remains undetected even though it affects patients with advanced liver disease. Unlike other Cardiomyopathies, which are associated with systemic diseases, CCM is uniquely characterized by its pathogenesis rooted in portal hypertension-induced autonomic and ion channel dysfunction [1]. According to research, the primary hepatic effects of cirrhosis extend to broader systemic involvement, including cardiac dysfunction [1]. The ECG readings of patients with CCM show three distinct patterns, including prolonged QT intervals, elevated QRS voltages, and particular T-wave abnormalities [2]. The markers allow doctors to identify the disease using tests that do not require invasive procedures. The detection of ECG changes in CCM is crucial for enhancing diagnostic accuracy and optimizing therapeutic outcomes [3].

The pathophysiological mechanisms of CCM include autonomic dysfunction, myocardial remodeling, and alterations in ion channel activity. The study of QT prolongation is a prominent research topic because it demonstrates ventricular dysfunction. The severity of liver disease shows a direct relationship with QT prolongation, as indicated by both the Child-Pugh classification and the Model for End-Stage Liver Disease (MELD) score [3,4]. QT prolongation is found in all stages of cirrhosis, particularly in advanced stages of the disease [4]. QRS voltage and T-wave changes, however, have been found to differ in some populations, which can be due to variability in fluid status, fibrosis, or metabolic changes. The combination of gastrointestinal bleeding and liver transplantation as acute clinical stressors leads to QT prolongation, which increases the risk of ventricular arrhythmia [5]. The ECG feature of CCM includes QRS hyper-voltage as a distinct characteristic from the QT interval. The condition results in myocardial fibrosis, metabolic disturbances, and fluid redistribution [6]. The electrophysiological abnormality in cirrhotic patients is demonstrated by T-wave morphology and prolonged T-peak to T-end intervals [7]. Despite widespread documentation of ECG abnormalities in cirrhosis, their correlation with myocardial structural changes remains uncertain. For example, Toma et al. observed QT prolongation to be independent of structural myocardial alterations [3], whereas other studies linked QT changes to extracellular volume expansion. These variabilities show the need for standardized ECG protocols. Ventricular function evaluated using echocardiography in cirrhotic patients has given mixed results regarding the diagnostic value of ECG markers [8]. Some studies suggest that QT prolongation may not be a definite marker for CCM-related myocardial remodeling. It functions as a separate electrophysiological entity linked to cirrhosis [4]. Research findings reveal decreased ejection fraction and diastolic dysfunction, while underscoring the importance of combined diagnostic methods [4].

The determination of ECG markers for CCM can enhance the accuracy of diagnosing and treating patients with CCM, and enable physicians to assess and treat cardiac complications more effectively [9]. Considering the heterogeneity in ECG changes and cardiac dysfunction in patients with cirrhosis, future research should be directed toward combining echocardiography and autonomic function testing. These strategies are not standardized at this time; however, they have the potential to be useful in the early identification of CCM [3]. Researching the progression of ECG changes in cardiomyopathy can further this understanding of CCM pathophysiology and prognosis [10]. In addition, investigations into biomarkers such as NT-pro BNP and cardiac troponins have increased the likelihood of improving CCM diagnostic criteria [11].

This study aims to summarize the current understanding of CCM, emphasizing its ECG markers and their diagnostic relevance. It examines cardiac dysfunction in patients with liver disease by reviewing the changes in QT interval prolongation, T-wave morphology, and QRS voltage. Physicians can optimize care in CCM by using additional diagnostic methods beyond ECG evaluation, such as echocardiography and cardiac biomarkers. This multimodal approach acknowledges the complexity of myocardial remodeling in cirrhosis and improves diagnostic accuracy.

## Review

Methods

We conducted this systematic review according to the 2020 guidelines of the Preferred Reporting Items for Systematic Reviews and Meta-Analyses (PRISMA) [12].

Inclusion and exclusion criteria

In this systematic review, we included papers published in the last 10 years for clinical relevance and methodological rigor, written in English, and with full texts available to ensure accuracy. Articles focusing on human studies with participants diagnosed with cirrhosis from various causes were considered. Studies published more than 10 years ago, in languages other than English, or non-human studies were excluded from the review due to their insufficient relevance and potential for bias. Additionally, participants without a diagnosis of cirrhosis were omitted. Abstracts, posters, and conference presentations without corresponding full-text articles were also excluded, as they lacked sufficient details. The summary of the inclusion and exclusion criteria is listed in Table 1.

**Table 1:** Summary of the inclusion and exclusion criteria for the selected studies

Conditions	Inclusion	Exclusion
Publication year	Last 10 years	Over 10 years
Language	English	Languages other than English
Full-text access	Available	Not available
Study type	Human studies with diagnosed cirrhosis	Non-human studies due to insufficient relevance and potential bias
Diagnosis	Participants diagnosed with cirrhosis from various causes	Participants without a cirrhosis diagnosis
Publication type	Peer-reviewed articles with full-text access	Abstracts, posters, and conference presentations without full-text

Databases and search strategy

To identify relevant articles, we conducted a literature search from January 1, 2015, to May 1, 2025, using various databases, including PubMed, PubMed Central, ScienceDirect, Europe PubMed Central, and Google Scholar. We used keywords such as cirrhosis, liver dysfunction, decompensated liver disease, cardiomyopathy, cardiac dysfunction, ECG changes, diagnosis, and prognosis to develop our search strategy. Additionally, citations were also searched manually. All co-authors contributed actively to the creation of this systematic review. The screening was done independently by multiple reviewers. Inter-rater reliability was assessed informally through discussions, and formal kappa statistics were not calculated. We collaborated to collect data and refine the selection process for critically evaluating the quality of the chosen articles. Weekly Zoom sessions were conducted to discuss progress, share insights, propose improvements, and evaluate findings. This methodological approach significantly refined the search strategy and enhanced the overall rigor of the review. Additionally, final studies were discussed with co-authors during the Zoom sessions, and any disagreements were resolved. The detailed search strategy is explained in Table 2.

**Table 2:** Summary of the search strategy for each database MESH: Medical Subject Headings

Databases	Keywords	Search Strategy	Filters	Search result
PubMed	Electrocardiogram, Cirrhosis, Cardiomyopathy	"Liver Cirrhosis/classification"[Mesh] OR "Liver Cirrhosis/complications"[Mesh] OR "Liver Cirrhosis/diagnosis"[Mesh] OR "Liver Cirrhosis/etiology"[Mesh] OR "Liver Cirrhosis/pathology"[Mesh] OR "Liver Cirrhosis/physiopathology"[Mesh] )"Cardiomyopathies/classification"[Mesh] OR "Cardiomyopathies/complications"[Mesh] OR "Cardiomyopathies/diagnosis"[Mesh] OR "Cardiomyopathies/etiology"[Mesh] OR "Cardiomyopathies/pathology"[Mesh] OR "Cardiomyopathies/physiopathology"[Mesh] OR "Cardiomyopathies/prevention and control"[Mesh] )"Electrocardiography/classification"[Mesh] OR "Electrocardiography/instrumentation"[Mesh] OR "Electrocardiography/standards"[Mesh] OR "Electrocardiography/statistics and numerical data"[Mesh] OR "Electrocardiography/trends"[Mesh] )((( "Liver Cirrhosis/classification"[Mesh] OR "Liver Cirrhosis/complications"[Mesh] OR "Liver Cirrhosis/diagnosis"[Mesh] OR "Liver Cirrhosis/etiology"[Mesh] OR "Liver Cirrhosis/pathology"[Mesh] OR "Liver Cirrhosis/physiopathology"[Mesh] )) AND ( "Cardiomyopathies/classification"[Mesh] OR "Cardiomyopathies/complications"[Mesh] OR "Cardiomyopathies/diagnosis"[Mesh] OR "Cardiomyopathies/etiology"[Mesh] OR "Cardiomyopathies/pathology"[Mesh] OR "Cardiomyopathies/physiopathology"[Mesh] OR "Cardiomyopathies/prevention and control"[Mesh] )) AND ( "Electrocardiography/classification"[Mesh] OR "Electrocardiography/instrumentation"[Mesh] OR "Electrocardiography/standards"[Mesh] OR "Electrocardiography/statistics and numerical data"[Mesh]OR "Electrocardiography/trends"[Mesh] )	Free full text, From 2010 to 2025, English, Human studies, Preprints Excluded	48
Google Scholar	ECG Changes, Liver Cirrhosis, Decompensated Liver disease, Cardiomyopathy.	“ECG Changes” AND "Liver Cirrhosis" OR "Decompensated Liver Disease" AND "Cirrhotic Cardiomyopathy"	Free Full text, From January 1, 2015, to May 1, 2025, English, Human Studies, Preprints Excluded	2
PubMed Central Advanced	ECG Changes, Liver Cirrhosis, Decompensated Liver Disease, Cardiomyopathy.	(Electrocardiogram) AND (cirrhotic Cardiomyopathy)	Free Full text, From January 1, 2015, to May 1, 2025, English, Human Studies, Preprints Excluded	362
ScienceDirect	ECG Changes, Liver Cirrhosis, Decompensated Liver Disease, Cardiomyopathy.	"Electrocardiogram" and "Cirrhotic Cardiomyopathy"	Free Full text, From January 1, 2015, to May 1, 2025, English, Human Studies, Preprints Excluded	36
Europe PubMed Central	ECG Changes, Liver Cirrhosis, Cardiomyopathy	“Electrocardiogram OR ECG” AND “liver Cirrhosis OR Decompensated liver Disease” AND “Cardiomyopathy OR Cardiac Dysfunction”	Free Full text, From January 1, 2015, to May 1, 2025, English, Human Studies, Preprints Excluded	50

Search strategy

We gathered all relevant articles in EndNote (Clarivate, London, UK) and Rayyan AI (Rayyan Systems Inc., Cambridge, MA, US) and removed all the duplicates. These tools were primarily used to remove duplicates alone. Each article was reviewed initially for its title and abstract. After identifying suitable studies, we searched for free, full-text articles. The selected studies that met the inclusion and exclusion criteria were critically appraised for relevance. Finally, the selected articles that met all the necessary conditions were incorporated into the study.

Quality appraisal and data collection process

We used relevant quality appraisal tools to evaluate each selected Study based on its specific study design. We used the Newcastle-Ottawa scale [13] to assess observational studies and the Scale for the Assessment of Narrative Review Articles (SANRA) tool for narrative reviews [14]. Additionally, A Measurement Tool to Assess systematic Review (AMSTAR) 2 [15] was used for systematic reviews. The main objective of this research was to identify ECG changes in patients with liver cirrhosis and assess their effectiveness in detecting cardiomyopathy.

Results

The study began by searching multiple databases, including PubMed, which yielded 48 results; Google Scholar, which provided two results; PubMed Central, which returned 362 results; ScienceDirect, which returned 36 results; and Europe PubMed Central, which showed 50 results. Eight of the 498 articles were identified as duplicates, leaving 490 for title and abstract screening. After the screening process, 466 articles were excluded as they did not meet the inclusion criteria. The remaining 24 articles were retrieved. Three articles couldn't be retrieved, leaving 21 full-text articles for eligibility screening. Upon reviewing the 21 full-text articles, 13 were excluded due to their poor methodological quality. This process yielded eight articles that satisfy the inclusion criteria for the systematic review. These articles were evaluated using tools such as AMSTAR 2, the Newcastle-Ottawa Scale, SANRA, and the Joanna Briggs Institute (JBI) critical Appraisal Checklist [16].

The selected eight articles included a variety of designs, which are summarized in Table 3.

**Table 3:** Summary of the final studies and their type and appraisal tool AMSTAR 2: A Measurement Tool to Assess systematic Review 2; SANRA: Scale for the Assessment of Narrative Review Articles

Study type	Number of studies	Appraisal tool
Non-randomized controlled trials	6	Newcastle-Ottawa Scale (NOS)
Systematic review and meta-analysis	1	AMSTAR 2
Narrative literature	1	SANRA

**Figure 1:**
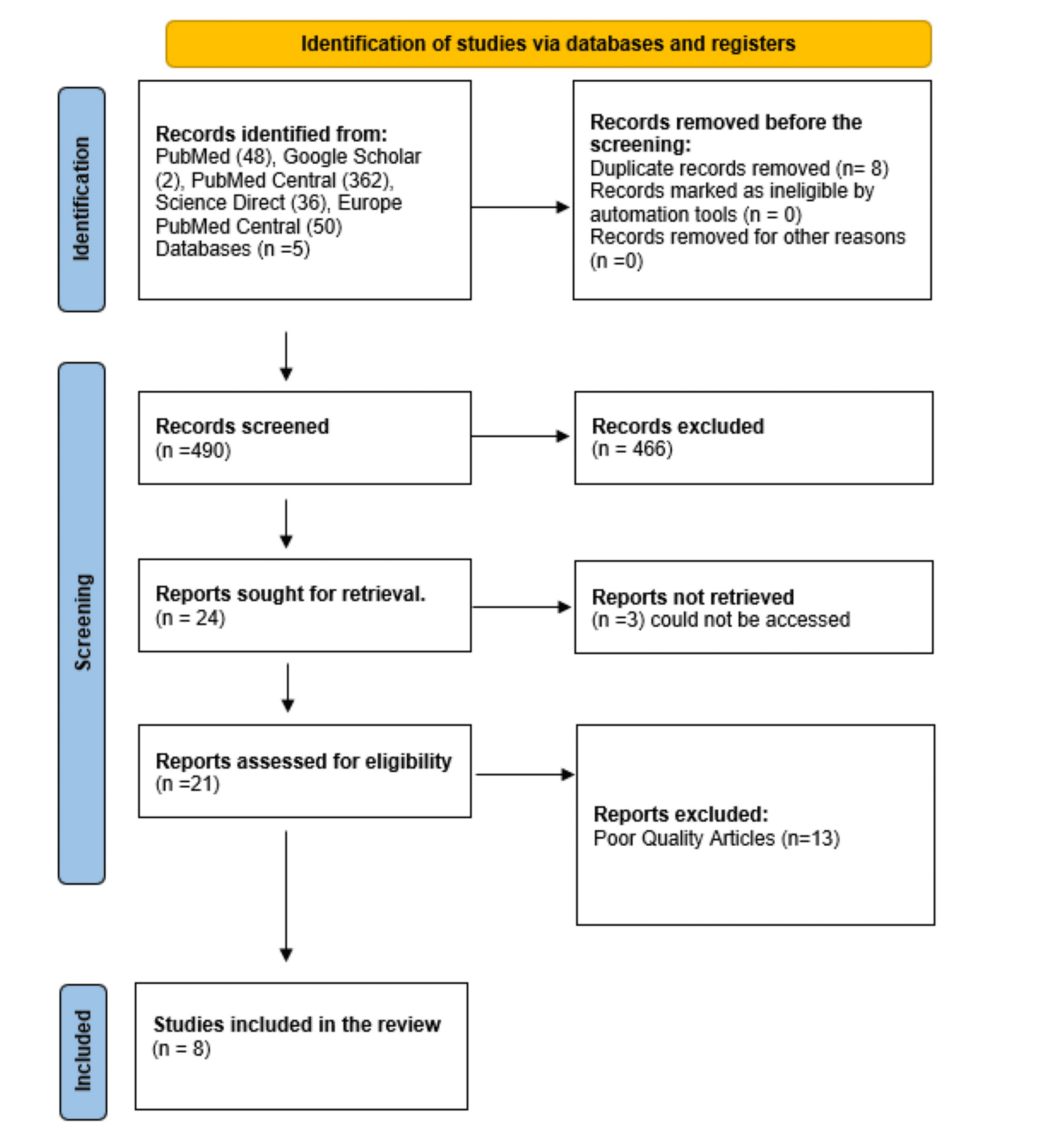
The flowchart represents the articles included in this review as per the PRISMA guidelines PRISMA: Preferred Reporting Items for Systematic Reviews and Meta-Analyses. The flowchart explains the number of records identified, screened, excluded, and included in the study.

These included retrospective and prospective observational studies, narrative reviews, and one systematic review. All the included studies were assessed to identify ECG changes in patients with cirrhosis and their potential role in CCM. There was considerable variation in study designs, populations, and outcomes; therefore, the data were synthesized descriptively rather than through meta-analysis. The entire process of selection is shown in below (Figure 1).

This review protocol was not registered on International Prospective Register of Systematic Reviews (PROSPERO). The decision was based on the exploratory nature of the study and the limited number of eligible studies available at that time.

Study characteristics

The study characteristics of the selected articles are summarized in Table 4.

**Table 4:** The main characteristics of the included articles are listed in the table MELD, Model for End-Stage Liver Disease; EF, Ejection Fraction; Tpe, T-peak to T-end interval.

Author Name and year	Study type	Total participants	Inclusion and exclusion criteria	Outcomes & key findings	Funding
Carvalho et al., 2019 [1]	Narrative review	N/A	Included studies discussing cirrhotic cardiomyopathy focusing on pathophysiology, diagnosis, and treatment. Excluded abstracts, letters to editors, non-English articles	Cirrhotic cardiomyopathy (CCM) involves ventricular dysfunction and QT interval prolongation. Pathogenesis involves altered beta-receptor function, excessive nitric oxide production, and impaired transmembrane ion currents. Liver transplantation may improve CCM, but the prognosis remains uncertain.	Not stated
Toma et al., 2020 [3]	Retrospective Observational Study	117	Included cirrhotic patients and chronic hepatitis patients; Excluded those with prior cardiovascular disease, arrhythmias, diuretic or antiarrhythmic use	QT prolongation correlated with liver disease severity, QRS voltage was lower in decompensated Cirrhosis, and Tpe interval shortening was observed. Findings suggest ECG is a useful noninvasive CCM marker.	Not Stated
Koshy et al., 2021 [7]	Retrospective Observational Study	439	Included patients undergoing liver transplant work-up; Excluded those with prominent coronary artery disease, valvular heart disease, or preexisting cardiomyopathy.	There is no noteworthy association between QT prolongation and myocardial remodeling, systolic function, diastolic function, cardiac reserve, or CCM criteria. Suggests QTc prolongation is independent of CCM structural markers	National Health and Medical Research Council of Australia; National Heart Foundation Post-Graduate Scholarship; Royal Australasian College of Physicians Blackburn Scholarship
Dourakis et al., 2021 [17]	Narrative Review	N/A	Reviewed literature on hemodynamic alterations, electrophysiological abnormalities, and myocardial dysfunction in cirrhosis	CCM results from hyperdynamic circulation, impaired myocardial contractility, diastolic dysfunction, and QT prolongation. Emphasizes diagnostic challenges and therapeutic gaps in CCM management	Not stated
Papadopoulos et al., 2023 [18]	Systematic Review & Meta-analysis	14,495	Included studies on QT prolongation in cirrhosis (1998 onward); Excluded case reports, reviews, abstract-only publications	QT is consistently prolonged in cirrhosis, correlates with disease severity, improves post-transplant, and is influenced by beta-blockers and acute illness. QT>440 ms is associated with a shortened survival.	Not stated
Bernardi et al., 2012 [19]	Narrative Review	N/A	Examines QT interval abnormalities in cirrhosis, focusing on epidemiology, molecular mechanisms, prognostic relevance, and clinical impact	QT prolongation is a hallmark of CCM, with an elevated risk of ventricular arrhythmias and sudden cardiac death. Investigates the role of cardiotoxins and sympathetic nervous system activation	Not stated
Gollamudi et al., 2024 [20]	Cross-Sectional Study	50	Included hospitalized cirrhotic patients; Excluded patients under 18, those with COPD, or preexisting heart disease.	QT prolongation correlates with Child-Pugh and MELD scores, with severe liver impairment linked to QT >440 ms and EF≤ 55%. Larger right and left atrial sizes were observed in Class C patients, with continuous cardiac monitoring emphasized for those with cirrhosis.	Not stated
Kim et al., 2020 [21]	Prospective Observational Study	439	Included cirrhotic patients awaiting liver transplant; Excluded those with existing cardiac disease	QT prolongation correlated with extracellular volume fraction (ECV) expansion, suggesting diffuse myocardial fibrosis. QT interval normalized post-transplant, reinforcing the reversibility of cirrhotic myocardial dysfunction.	Not stated

The remaining 13 articles were rejected due to poor quality and failure to meet appropriate methodological standards. These are summarized in Table 5.

**Table 5:** Summarizes the articles of low/moderate quality that were rejected

First author	Type of study	Quality appraisal tool & score	Quality score
Abdu et al. [22]	Retrospective observational study	Newcastle-Ottawa Scale (NOS)	3/9
Šimić et al. [23]	Literature review	Critical Appraisal Skills Program (CASP)	Low
Shahvaran et al. [24]	Systematic review & meta-analysis	A Measurement Tool to Assess systematic Reviews (AMSTAR) 2	10/16
Kumar et al. [25]	Cross-sectional study	Newcastle-Ottawa Scale (NOS)	3/9
Lee et al. [26]	Review	Critical Appraisal Skills Program (CASP)	Low
Fede et al. [27]	Review	Critical Appraisal Skills Program (CASP)	Low
Bhardwaj et al. [28]	Observational study	Newcastle-Ottawa Scale (NOS)	4/9
Sezgin et al. [29]	Observational study	Newcastle-Ottawa Scale (NOS)	6/9
Naqvi et al. [30]	Prospective analytical study	Newcastle-Ottawa Scale (NOS)	5/9
Lupu et al. [31]	Prospective observational study	Newcastle-Ottawa Scale (NOS)	2/7
Lu et al. [32]	Observational study	Newcastle-Ottawa Scale (NOS)	5/9
Țieranu et al. [33]	Observational study	Newcastle-Ottawa Scale (NOS)	6/9
Tsiompanidis et al. [34]	Cross-sectional study	Newcastle-Ottawa Scale (NOS)	5/9

A low, moderate, or high score indicates that the studies have not met the standard methodological requirements, including reliability, internal validity, and low risk of bias. The thresholds applied for tools include AMSTAR 2 (≥12/16), NOS (≥6/9), and Cochrane (≥4/7). Articles scoring equal to or below this threshold were considered poor quality and excluded from the study. In contrast, articles scoring above this threshold were considered of good quality.

Discussion

ECG Markers in CCM: Noninvasive Detection and Prognostic Value

CCM is a serious condition that impacts the heart function in liver disease, but it is often overlooked [35]. Since it frequently goes undiagnosed, treatment is mainly focused on cirrhosis and its complications and not on heart-related effects [1]. Cirrhosis initially targets the liver, yet results in systemic consequences that produce myocardial dysfunction in the heart [17]. In noninvasive markers for CCM, ECG changes stand out as a promising option [2]. Research continues to investigate the extent to which these changes impact structural and functional cardiac alterations. ECG abnormalities serve as early signals of myocardial involvement in cirrhotic patients before any structural changes become apparent. Despite the necessity of echocardiography and biomarker assessments as diagnostic tools, ECG findings present an affordable and readily available screening approach to identify possible cardiac dysfunction in patients with cirrhosis [3].

**Figure 2:**
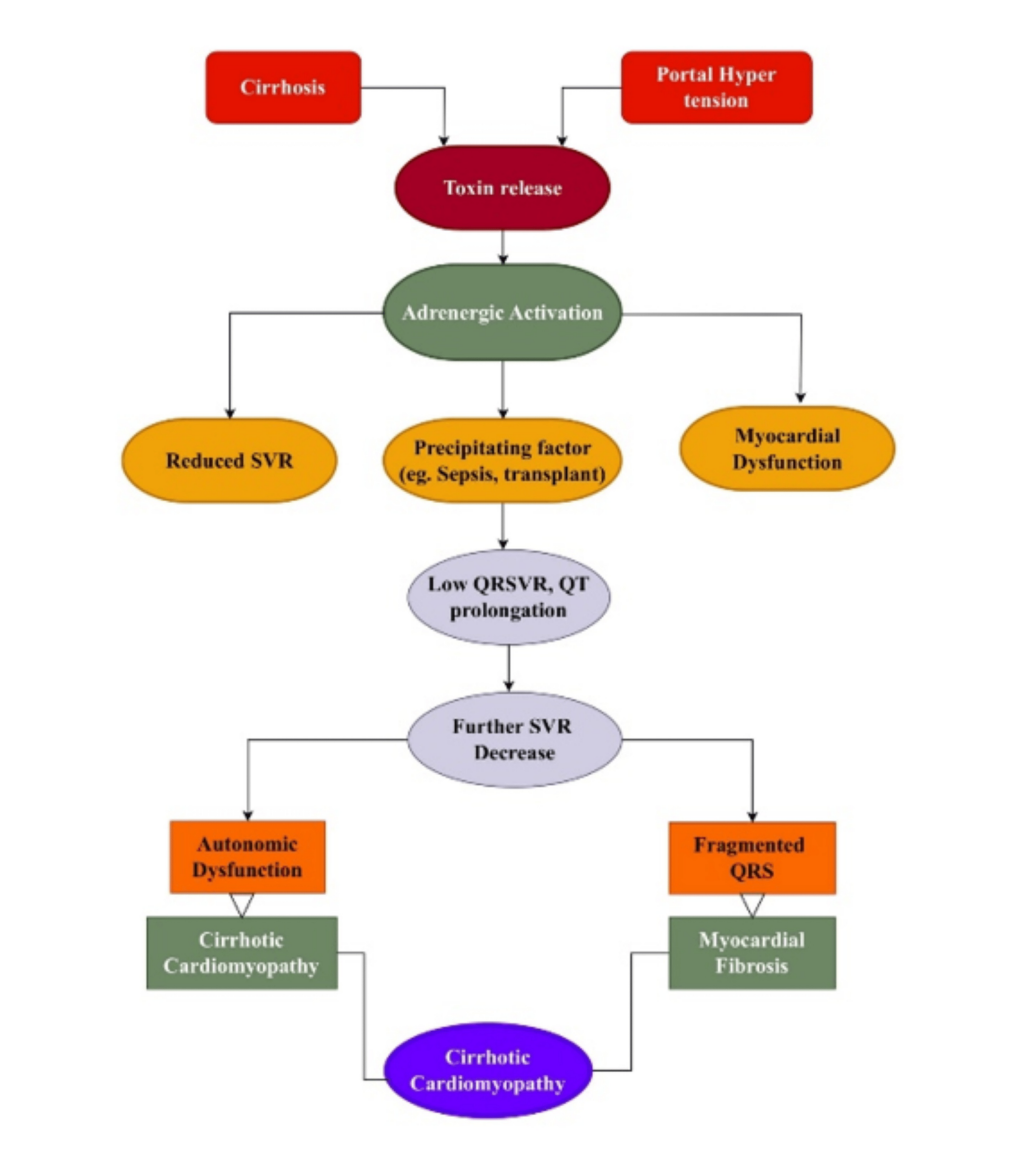
An outline of the interconnected pathophysiological mechanisms, autonomic dysfunction, ion channel abnormalities, and myocardial remodelling, that lead to cirrhotic cardiomyopathy in patients with liver cirrhosis SVR: Systemic Vascular Resistance; Flowchart created by the first author, SNR, with Draw.io (Jgraph Ltd, UK).

This discussion explores the diagnostic and prognostic significance of ECG changes in CCM. The study emphasizes the need for an integrated approach in cardiovascular assessments in patients with cirrhosis by examining the pathophysiology behind this disease and its relationship with the ECG findings. The pathophysiological outline of CCM and associated ECG changes is presented in Figure 2 as follows.

Pathophysiological Mechanisms: Autonomic Dysfunction, Ion Channel Dysregulation, and Myocardial Remodeling

**Figure 3:**
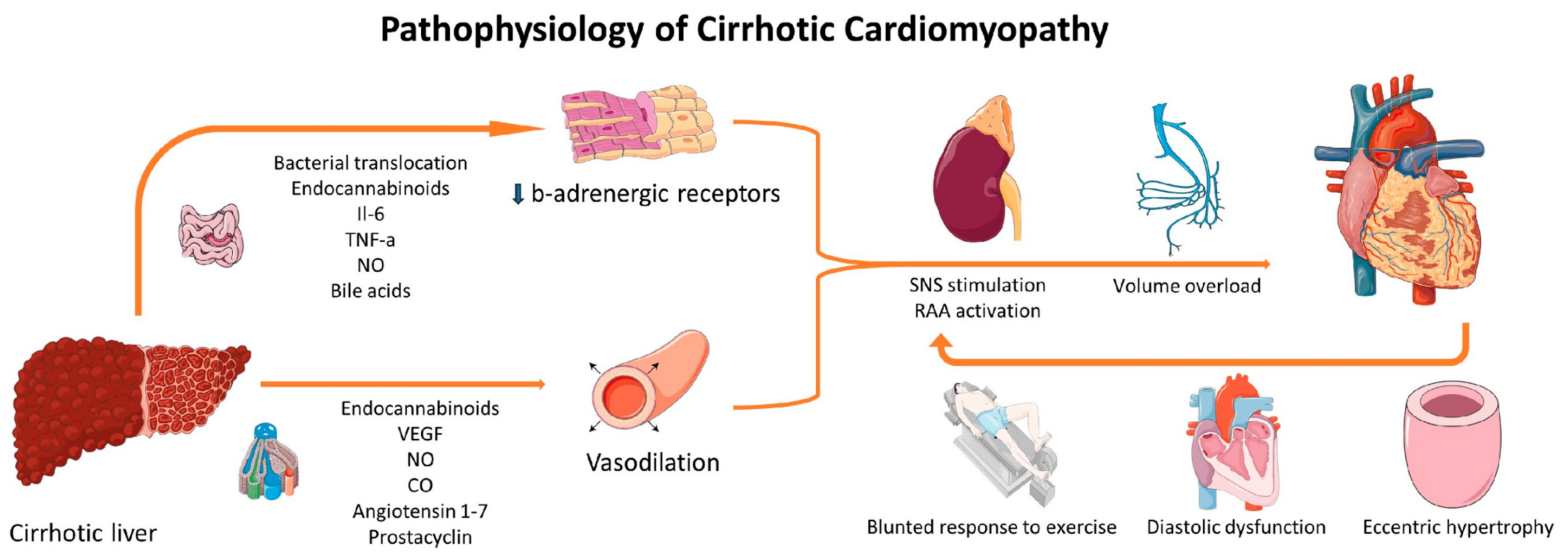
The sequential events triggered by portal hypertension, leading to autonomic activation, elevated cardiac output, and myocardial remodeling, cumulatively leading to electrophysiological abnormalities CO: carbon monoxide; IL-6: interleukin-6; NO: nitric oxide; RAAS: renin-angiotensin-aldosterone system; SNS: sympathetic nervous system; VEGF: vascular endothelial growth factor. [36]

Cirrhosis has a wide range of effects on heart function, leading to various electrical and structural changes contributing to CCM. Several key interconnected mechanisms explain the alterations seen in ECG readings. The key pathophysiological mechanisms of CCM are represented in Figure 3.

Autonomic dysfunction and its role in ECG abnormalities: The autonomic system regulates cardiac function. However, cirrhosis creates excessive sympathetic activity and a shutdown of the parasympathetic system [18]. This shuts off the normal heart rhythm and makes the heart electrically unstable. Catecholamines cause overcrowding of the cardiac muscle and delay ventricular repolarization, which lengthens the QT interval [37]. A diminished vagal tone produces repolarization irregularities that increase the risk of dangerous arrhythmias, including torsades de pointes [37].

Ion channel dysregulation: CCM is also characterized by ion channel dysfunction, which causes irregular heart activity. The dysfunction in ion channels that cause the ECG abnormalities found in CCM include a decreased efflux of potassium ions during repolarization, which prolongs the action potential and causes QT abnormalities [38]. Additionally, calcium cycling in cardiac myocytes is disrupted, resulting in decreased cardiac contractility and mechanical and electrical instability [39]. Lastly, an interruption of sodium and potassium flow prolongs the repolarization period, sensitizing the heart to arrhythmias [38].

Myocardial remodeling and fibrosis: The changes due to cardiac cirrhosis are permanent and progressive over time, leading to distortion in ventricular contraction. Myocardial remodeling alters the heart's function, which is triggered by chronic systemic inflammation, excessive nitric oxide production, and hormonal imbalances [18]. These effects can be explained by ECG changes, specifically altered QRS voltage, resulting from structural heart abnormalities [15].

Multimodal ECG Assessment in CCM: ECG, Echocardiography, Biomarkers, and AI-Driven Insights

ECG serves as a valuable tool in identifying cardiac conduction abnormalities related to CCM. Several key findings have been observed.

QT interval prolongation: Studies estimate that QT interval prolongation appears in 40-50% of CCM cases when analyzing ECG abnormalities [1].

**Figure 4:**
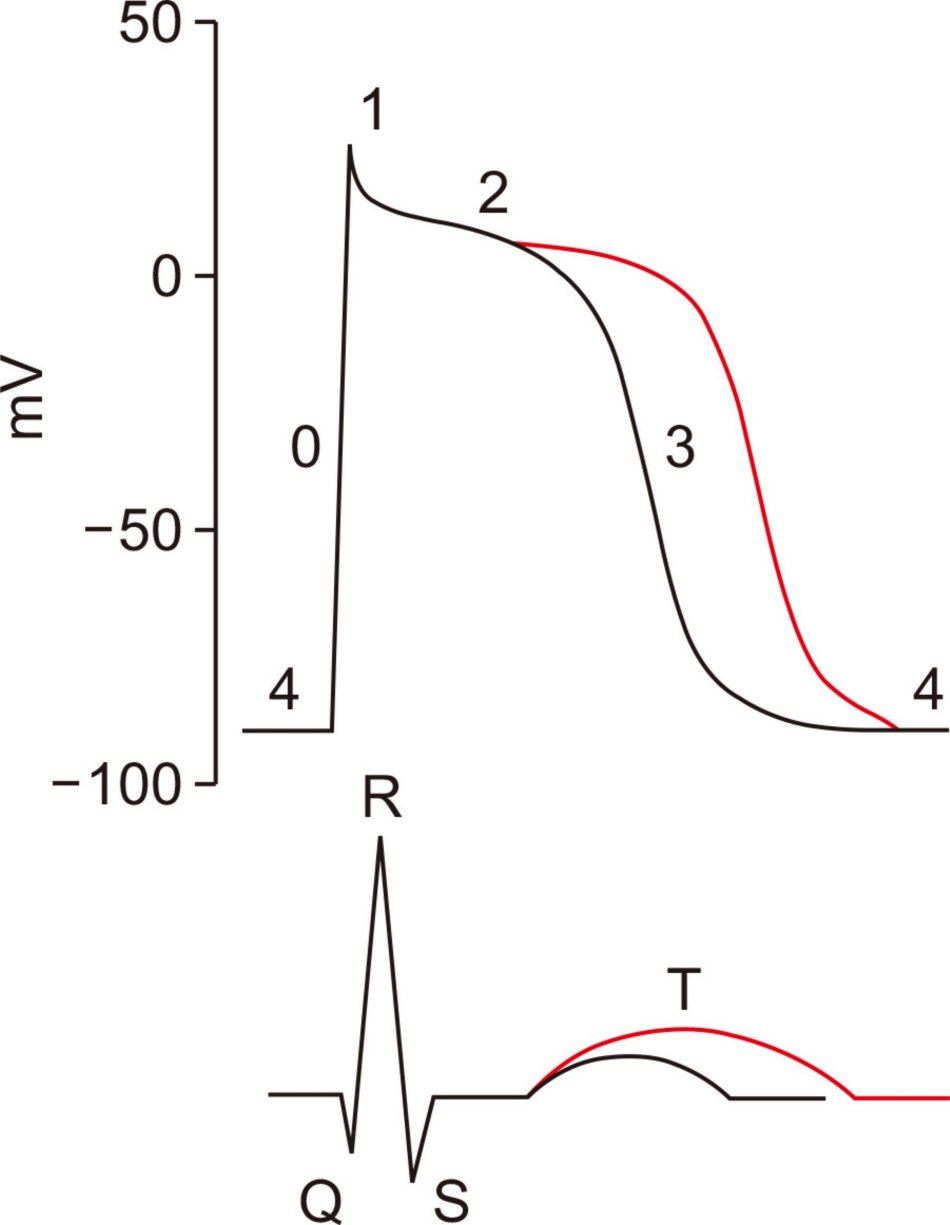
The figure compares a standard ventricular action (black line) with a prolonged action potential (red line), highlighting the electrophysiological abnormality in patients with cirrhotic cardiomyopathy [26]

**Figure 5:**
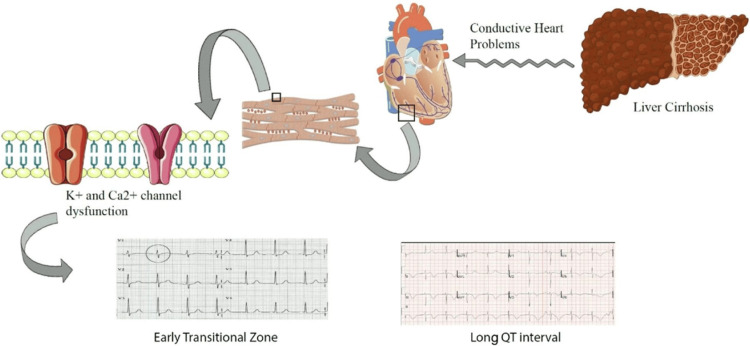
This figure illustrates how elevated portal pressure and systemic inflammation contribute to myocardial remodeling and QT interval prolongation, further supporting the findings of how liver disease affects the cardiac conductive system [40]

Clinical significance and association with cirrhosis severity: It is important to note that patients with prolonged QT Intervals have a higher risk of developing arrhythmias such as torsades de pointes. The subsequent arrhythmias result in fatal events and complications such as syncope, as well as sudden cardiac death, and cardiogenic shock [5]. This explains the importance of constant monitoring and early detection in patients with cirrhosis. Additionally, QT prolongation correlated with the severity of liver disease. The electrical instability in the heart worsens as Child-Pugh and MELD scores worsen, further increasing the risk of cardiac complications [20]. This further solidifies the need for routine ECG monitoring in patients with cirrhosis. The findings of QT prolongation and the pathophysiological mechanism for the association between ECG changes and myocardial fibrosis are shown in Figures 4 and Figure 5 respectively.

QRS voltage changes and cardiac remodeling: The QRS voltage alterations are often associated with myocardial remodeling and reflect the impact of x=cirrhosis on cardiac function. QRS hyper voltage may indicate that the heart adapts to stress, leading to myocardial hypertrophy [3]. Similarly, low QRS voltage is often associated with fluid retention, hepatic congestion, and metabolic derangements [21]. Dimitroglou et al. (2019) suggested that myocardial remodeling and fibrosis play a significant role in CCM; however, the ECG findings regarding QRS voltage and structural cardiac changes vary [8]. These inconsistencies suggest that QRS hyper-voltage does not universally represent myocardial fibrosis. Other factors like fluid redistribution, metabolic factors, and technical variability in ECG interpretation can also result in this variations.

T-wave morphological changes and prognostic implications: T-wave morphological changes indicate a disruption in the heart's electrical signal functions, often pointing to early signs of heart failure [3]. Studies suggest that prolonged T-peak to T-end intervals result in greater instability [21]. Patients with cirrhosis face an elevated risk of arrhythmia and other critical cardiac events due to this condition [1,21]. The simultaneous presence of T-wave abnormalities with QT prolongation indicates an early signal of deteriorating heart function [41]. A heart health evaluation should integrate ECG results with additional diagnostic techniques to ensure a thorough assessment. The method provides reliable assessments of heart function alongside precise monitoring of patient prognosis.

Clinical Relevance

As discussed, our systematic review on ECG abnormalities can be understood as a surrogate for early diagnosis of CCM. The significantly elevated prevalence of QT prolongation, QRS voltage changes, and T-wave changes among patients with liver cirrhosis has demonstrated that implementing a simple screening ECG for the routine assessment of cardiac dysfunction before clinical manifestation is more plausible. On the other hand, between-study variation of ECG changes in terms of prevalence was found to be high, and the contributing factors were mainly due to differences in patient selection, cirrhosis severity, ECG protocol, and lack of set standard criteria [3,7,20].

QT prolongation was found to be associated with the severity of liver disease. As Child-Pugh and MELD scores decline, electrical instability in the heart is further elevated, leading to higher risks of cardiac complications [5,7,20]. Although QT prolongation was found to be a prevalent, this marker is not specific when compared to dedicated biomarkers, such as NT-proBNP. Sensitivity for detecting cardiac dysfunction is improved when a combination of ECG and biochemical markers are used [8,11]. QTc prolongation should be carefully interpreted in clinical practice because the impact of medications (e.g., beta-blockers, diuretics) and electrolyte disturbances on prolongation can overlap with CCM. In some studies, QTc prolongation has been adjusted for these variables, while some other studies have excluded patients using QTc-prolonging medications [7,19].

Combining ECG data with echocardiography and cardiac biomarkers leads to a more robust risk assessment. While ECG changes can precede biomarker elevation and indicate early electrophysiological instability, the integration of both modalities can improve prognostic value and risk stratification. This approach can be particularly beneficial for patients who are potential candidates for liver transplantation [4,11].

Optimization of perioperative management and long-term care is also well-structured by AI-assisted or multimodal diagnostic approach, and this can also be effective for CCM. Despite the potential of AI-assisted ECG diagnosis, some of the main challenges for this application have not been solved yet. These include issues with inconsistent or low-quality data, lack of external validation or robust performance on independent datasets, and limited integration into clinical workflows and electronic medical records (EMRs). Interestingly, most studies described a possible application of AI but discussed this more on a speculative level, and no validated and CCM-specific models have been presented yet [42].

The field of AI in healthcare has been experiencing an exponential growth in the recent years with the introduction of advanced models in various clinical settings, promising to provide greater diagnostic accuracy and earlier intervention measures for various conditions. Standardized protocols for ECG interpretation using AI applications can reduce the interpretational variability between physicians, leading to more consistent and reliable evaluation of patients. Moreover, these methods are now more likely to lead to better patient care in terms of health outcomes and mortality rates, specifically among patients with cirrhosis.

In view of current evidence, it might be feasible to recommend an ECG screening for cirrhotic patients in the future guidelines in those with advanced disease (Child-Pugh B/C) or those who are on a transplant waiting list, or requiring more outcome-based validation studies [7,20]. This potential recommendation was emphasized by studies like Koshy et al. and Gollamudi et al., who related the ECG abnormalities to overall survival and transplant eligibility, which further supports the prognostic use of ECG in clinical decision-making [7,20].

Future Directions

Future studies of CCM should determine more objective diagnostic criteria, improve risk stratification, and discover mechanism-specific treatments. To progress further, ECG data can be further combined with echocardiography results, cardiac biomarkers, and autonomic testing. More prospective research is needed to broaden ECG use for the detection of cardiac issues, verify current findings, and reduce bias, thus improving the accuracy of the results. Additional research can be done on the evolution of ECG patterns throughout different life stages, which can help diagnose cardiac disease at earlier stages. Clinical guidelines should focus on the importance of regular cardiac screening for patients with cirrhosis, as CCM is often undiagnosed. ECG analysis is performed with more accuracy and efficiency with advancements in AI and machine learning. Healthcare professionals who prioritize regular cardiac monitoring for patients with cirrhosis experience better treatment outcomes.

Study Limitations

This study was not without limitations, and it is essential to consider these when interpreting the results. First, the small number of studies with limited sample sizes and the diversity of study designs of the included studies may impact the external validity of the current review. Second, the results could be inaccurate due to the heterogeneity of ECG measurement protocols and inconsistencies in diagnostic thresholds. The varied definitions of clinical remission and ECG abnormalities across the studies made interpretation and comparability challenging, which may affect the results of this systematic review. The cross-sectional nature of most of the studies included in the current systematic review limited our ability to evaluate the progression of ECG changes associated with CCM. The lack of consistent use of a control group in the included studies limited our ability to determine if the cardiomyopathy is a direct result of cirrhosis or the result of other cardiac abnormalities. ECG is a noninvasive monitoring of cardiac dysfunction, but the results of ECG in relation to echocardiography and biomarkers are inconsistent. Future research should focus on conducting large standardized studies over a longer duration to improve diagnostic accuracy and determine the best clinical practices. The detailed limitations of the study are listed in Table 6 below.

**Table 6:** The table represents the detailed limitations of the study

Limitation	Description	Impact on the Study
Limited number of high-quality studies	Only eight studies met inclusion and quality appraisal standards out of 498 screened.	Restricts the generalizability and depth of evidence synthesis.
Heterogeneity of study designs	Studies varied in design (e.g., retrospective, prospective, narrative review), populations, and outcome measures.	Prevented meta-analysis and made comparative analysis challenging.
Inconsistent ECG protocols	Variations in QT interval measurement methods, ECG equipment, and interpretation criteria were noted.	Introduced variability in ECG findings, affecting data consistency.
Lack of longitudinal data	Most included studies were cross-sectional or retrospective in design.	Limits understanding of ECG progression over time in CCM.
Absence of uniform diagnostic criteria for CCM	Different studies used varying definitions and thresholds for diagnosing CCM.	It makes it difficult to standardize findings and create unified recommendations.
Limited biomarker integration	Not all studies evaluated NT-proBNP, troponin levels, or echocardiographic correlates.	Reduces the ability to validate ECG findings with structural or biochemical evidence.
Potential publication bias	Studies with negative or inconclusive results may be underrepresented in the published literature.	Could overestimate the diagnostic value of ECG changes in CCM.
No meta-analysis performed	Due to study heterogeneity and small sample size, pooled statistical analysis was not feasible.	Limits the quantitative strength and estimation of effect size for ECG markers.

## Conclusions

The review of ECG abnormalities associated with CCM provides insights into their diagnostic and prognostic significance. Commonly reported ECG changes in CCM include QT prolongation, as noted in the analyzed studies. However, a pooled prevalence of QT prolongation could not be established due to the qualitative synthesis of the data. It is noteworthy that QT prolongation has recently been reported to independently predict poor outcomes in patients with cirrhosis, including arrhythmias and sudden cardiac death, rather than just reflecting the severity of the underlying liver disease.

The potential utility of NT-proBNP and troponins in the diagnosis of CCM is increasingly discussed. However, the cut-off levels for these biomarkers in the context of cirrhosis have not been established and validated. The integration of these biomarkers into a diagnostic algorithm for CCM warrants further prospective studies for confirmation.

AI-assisted ECG interpretation, as opposed to traditional ECG analysis, shows promise in enhancing diagnostic yield. It employs automated pattern recognition algorithms, minimizes inter-observer variability, and integrates multimodal data for more accurate interpretation. This approach may improve early detection and risk stratification in CCM, but its routine clinical implementation requires cost-benefit analysis and longitudinal studies correlating AI interpretations with patient outcomes.

The current evidence does not support routine ECG screening in patients with cirrhosis. Larger studies incorporating standardized outcome measures, such as arrhythmia events, transplant outcomes, and echocardiographic parameters, are necessary to establish the clinical utility of ECG in this population. The echocardiographic correlates of the ECG abnormalities, consistently observed across studies, include diastolic dysfunction, reduced ejection fraction, and myocardial fibrosis. These findings support the integration of cardiac assessment in patients with cirrhosis.

Clinical practice guidelines could consider insights such as a QTc interval >440 ms as a high-risk threshold, a T-peak to T-end interval prolongation as a marker of electrical instability, and QRS voltage changes in the context of myocardial remodeling, as implementing these changes may help refine diagnostic accuracy and inform risk stratification in CCM.
